# Melanin Deficiency Is Associated with Immune Homeostasis in the Critically Endangered Yangtze Sturgeon (*Acipenser dabryanus*)

**DOI:** 10.3390/ijms27125379

**Published:** 2026-06-15

**Authors:** Bin Wang, Yingzi Li, Han Sun, Fei Yang, Kezhen Jiang, Ya Li, Yixiao Xiong, Zhaoxiong Yu, Xueling Zhang, Peiqi Lv, Zhongliang Zhang, Xin Zhang, Zhiqiong Li, Bo Zhou, Ni Tang

**Affiliations:** 1 Fisheries Research Institute, Sichuan Academy of Agricultural Sciences (Sichuan Fisheries Research Institute), Chengdu 611731, China; wangbin@scsaas.cn (B.W.); yangfei27@scsaas.cn (F.Y.); yuzx0118@scsaas.cn (Z.Y.); zhang08042@scsaas.cn (X.Z.); 19982906619@163.com (P.L.); 18016198912@163.com (Z.Z.); zhoubo@scsaas.cn (B.Z.); 2 Department of Aquaculture, College of Animal Science and Technology, Sichuan Agricultural University, Chengdu 611130, China; 13350051633@163.com (Y.L.); sh17865265089@163.com (H.S.); dogan1012@163.com (K.J.); liya7911@163.com (Y.L.); xyx1233212023@163.com (Y.X.); 14769@sicau.edu.cn (X.Z.); 10986@sicau.edu.cn (Z.L.); 3 Fishes Conservation and Utilization in the Upper Reaches of the Yangtze River Key Laboratory of Sichuan Province, Fisheries Research Institute, Sichuan Academy of Agricultural Sciences (Sichuan Fisheries Research Institute), Chengdu 611731, China; 4 Fish Resources and Environment in the Upper Reaches of the Yangtze River Obervation and Research Station of Sichuan Province, Yibin 644000, China

**Keywords:** albinism, immune dysregulation, melanogenesis, skin, Yangtze sturgeon

## Abstract

The Yangtze sturgeon (*Acipenser dabryanus*), a critically endangered living fossil whose wild populations are now extinct, faces new challenges to survival in captive breeding. Among these, the emergence of albino and gray color morphs raise fundamental questions about the molecular basis and physiological consequences of pigmentation loss. Here, we integrated histological, transcriptomic, and quantitative PCR to investigate pigmentation variation and associated immune alterations in this species. Histology revealed a complete absence of melanin in albino individuals and marked reduction in gray morphs. Transcriptomic profiling across the three color morphs uncovered a broad downregulation of core melanogenic genes, including *PMEL*, *TYR*, *TYRP1*, *DCT*, *SLC45A2*, *OCA2*, *MREG*, and *MLPH*, indicating impaired melanosome formation, melanin synthesis, and intracellular transport. Notably, pigmentation loss coincided with systematic changes in the expression of immune-related genes: phagosome pathway genes (e.g., *C3*, *MHC I/II*, *TAP2*) were downregulated, while pro-inflammatory mediators (e.g., *IL-8*, *IL-17*, *CXCL10*) were upregulated, suggesting a transcriptional pattern correlated with reduced expression of pathogen defense-related genes and increased genes associated with inflammation mediators. These findings reveal a mechanistic correlation between melanin deficiency and immune dysfunction in a basal vertebrate lineage, offering the first molecular evidence of an association between albinism and altered immune-related gene expression in sturgeons and highlighting its implications for conservation and captive management.

## 1. Introduction

Body coloration is a fundamental and evolutionarily conserved trait that influences organismal fitness through its roles in camouflage, thermoregulation, mate selection, and environmental adaptation across vertebrates [1,2]. In aquatic environments, pigmentation serves a particularly critical ecological function, as visual predation exerts strong selective pressure on color patterning and melanin-based pigmentation provides protection against ultraviolet radiation and oxidative stress [3]. The synthesis and maintenance of melanin are governed by a highly coordinated molecular network centered on the microphthalmia-associated transcription factor (MITF), which regulates the key melanogenic enzymes tyrosinase (TYR), tyrosinase-related protein 1 (TYRP1), and dopachrome tautomerase (DCT), which operate within specialized organelles called melanosomes [4]. Although extensive research in mammals, reptiles, birds, and teleost model fishes has delineated the core pigmentation pathway [5,6,7,8], the degree to which these molecular mechanisms are evolutionarily conserved in basal vertebrate lineages remains largely unknown.

The Yangtze sturgeon (*Acipenser dabryanus*), an ancient “living fossil” belonging to the non-teleost ray-finned fish lineage (Chondrostei), provides a unique opportunity to investigate the ancestral state of vertebrate pigmentation biology [9]. This species diverged from the teleost radiation and thus retains many primitive morphological and physiological characteristics, making it an essential model for exploring pigment evolution in basal actinopterygians. However, its evolutionary significance coincides with a severe conservation crisis: the wild populations of *A. dabryanus* were officially declared extinct by the IUCN in 2022, and its survival now relies entirely on captive breeding programs [10,11,12]. The species historically inhabited the upper Yangtze River and its tributaries and, despite being a non-migratory freshwater sturgeon, could traverse distances exceeding 2000 km during its life cycle, facing multiple anthropogenic threats such as overfishing, vessel strikes, and habitat degradation [11].

Unexpectedly, recent breeding programs have reported the emergence of distinct pigmentation morphs, including albino (white) and gray variants, within captive *A. dabryanus* populations. This phenomenon presents both a unique research opportunity and a conservation challenge. The skin of sturgeons acts as a critical immunological and mechanical barrier against pathogens and environmental stressors [13], meaning that alterations in pigmentation could have profound physiological implications. Despite the well-established roles of genes such as *SLC45A2*, *OCA2*, and *HPS4* in albinism across derived teleosts, including the snakehead (*Channa argus*), goldfish (*Carassius auratus*), and channel catfish (*Ictalurus punctatus*), the molecular and functional basis of pigmentation loss in sturgeons remains unknown [14,15,16]. Moreover, beyond aesthetic variation, there is growing recognition that pigmentation defects may coincide with physiological impairments, particularly in immune competence, as melanin and melanogenic pathways intersect with redox balance, pathogen defense, and inflammatory regulation. Melanin can directly bind to bacterial surface components, generate reactive oxygen species, and modulate immune cell function, thereby contributing to innate antimicrobial defense. However, whether such links exist in ancient fish lineages remains an open and important question for both evolutionary biology and conservation physiology.

In this study, we integrate histological, transcriptomic, and quantitative gene expression analyses to investigate the cellular and molecular mechanisms underlying pigmentation loss in the Yangtze sturgeon and to assess its potential skin-associated molecular alterations. By comparing black, gray, and albino phenotypes, we examined structural changes in skin melanosomes, differential expression of canonical melanogenesis genes, and downstream alterations in immune-related pathways. Our findings provide the first comprehensive characterization of albinism in *A. dabryanus* and reveal an unanticipated connection between melanin deficiency and immune changes. These results not only expand our understanding of pigment biology in basal vertebrates but also offer critical insights into the management of genetic diversity and health in conservation breeding programs for this critically endangered species.

## 2. Results

### 2.1. Phenotypic Variation and Melanin Distribution Among Yangtze Sturgeon Color Morphs

**Figure 1:**
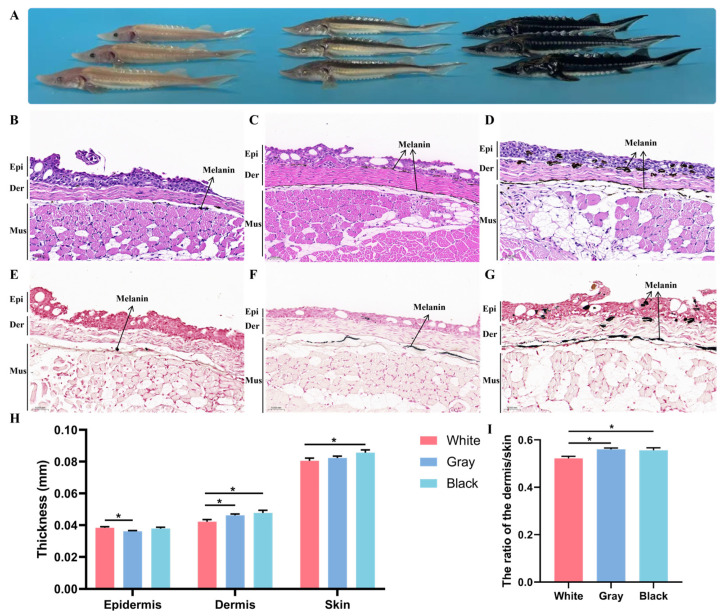
Phenotypes and histological features of black, gray, and white Yangtze sturgeons. (**A**): Representative photographs of the three color morphs. (**B**–**D**): Hematoxylin–eosin (H&E) staining of skin sections, scare bar 0.05 mm. (**E**–**G**): Fontana–Masson staining showing melanin deposition (black granules) across morphs, scare bar 0.05 mm. The major structural components are labeled: epidermis (Epi), dermis (Der), and muscle layer (Mus). (**H**): Comparison of the thickness of the epidermis, dermis, and skin (μm). (**I**): The ratio of dermis layer to intact skin of Yangtze sturgeon across morphs. *n* = 9 fish per group for histology. Asterisks indicate significant differences. * *p* < 0.05 indicates a statistically significant difference between the gray or black group and the white group, which were tested by one-way ANOVA with Tukey’s post hoc.

The juvenile Yangtze sturgeons exhibiting black, gray, and white color morphs showed no significant differences in either body weight or total length (black: 7.93 ± 0.49 g, 13.22 ± 0.49 cm; gray: 7.67 ± 0.94 g, 13.33 ± 0.94 cm; white: 7.45 ± 0.99 g, 12.85 ± 0.99 cm; *p* > 0.05). However, histological analyses revealed distinct epidermal and dermal structural organization among the morphs (Figure 1A).

We found that white individuals exhibited loosely arranged epidermal cells with larger intercellular spaces, reduced cellular density in the dermal loose layer, and sparsely distributed, morphologically simple mucous cells (Figure 1B). However, gray individuals showed moderate epidermal cell compaction, increased mucous cell abundance, and greater structural cohesion in both dermal layers (Figure 1C). The black individuals showed qualitatively the highest epidermal density, minimal intercellular spacing, and numerous, morphologically diverse mucous cells within the dermis, indicative of enhanced tissue organization and physiological activity (Figure 1D). The Fontana–Masson staining further revealed pronounced differences in melanin deposition among color morphs. Specifically, white individuals displayed a complete loss of melanin and absence of identifiable melanocytes, with their lack of melanin deposition confirmed by light microscopy (Fontana–Masson staining); this condition is referred to as phenotypically albino throughout this study (Figure 1E). In gray individuals, melanin granules were scattered and discontinuous, with moderate pigmentation limited to the epidermal layer (Figure 1F). Black sturgeons exhibited abundant, continuous melanin granules extending from the epidermis to the loose and dense dermal layers, forming a distinct dark pigment band at the epidermal–dermal junction (Figure 1G). The results demonstrated that overall skin thickness was significantly reduced in white morphs compared to black morphs. Moreover, skin thickness in white individuals was substantially lower than in both gray and black morphs (*p* < 0.05, Figure 1H). Additionally, the relative proportion of the dermal layer within the total skin thickness was significantly smaller in white morphs than in gray or black individuals (*p* < 0.05, Figure 1I).

### 2.2. Transcriptome Sequencing, Assembly, and Quality Assessment

**Figure 2:**
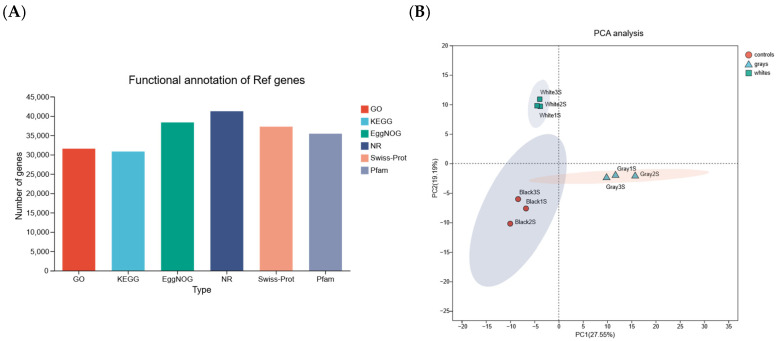
Transcriptome overview of Yangtze sturgeon skin. (**A**): Functional annotation summary across reference database. (**B**): PCA plot illustrating sample clustering and variance explained by principal component 1 (PC1) and 2 (PC2). The PCA plot is based on RNA-seq data from three pooled biological replicates per color morph (each pool = 3 fish). The *X*-axis and *Y*-axis, respectively, represent the contribution of PC1 and PC2 to the differentiation of samples.

RNA sequencing was performed on three pooled biological replicates per color morph (each pool containing skin from three fish), resulting in nine libraries in total. High-throughput RNA sequencing of nine skin samples (PRJNA1389687) generated a total of 54.14 Gb of clean reads, with each sample exceeding 5.46 Gb. The RNA Quality Number (RQN) values for all nine skin samples ranged from 8.3 to 9.7, indicating high RNA integrity, suitable for reliable transcriptome analysis. The sequencing quality was high, as indicated by an average error rate below 0.0128% and Q30 values exceeding 94.41% (Table 1). Transcriptome assembly yielded 128,921 transcripts, of which 93.66% (120,799) were successfully annotated against the NR database (Figure 2A). Principal component analysis demonstrated strong intra-group correlation and clear inter-group separation, with gray morphs clustering closer to black than white individuals (Figure 2B).

### 2.3. Differential Gene Expression Profiling

**Figure 3:**
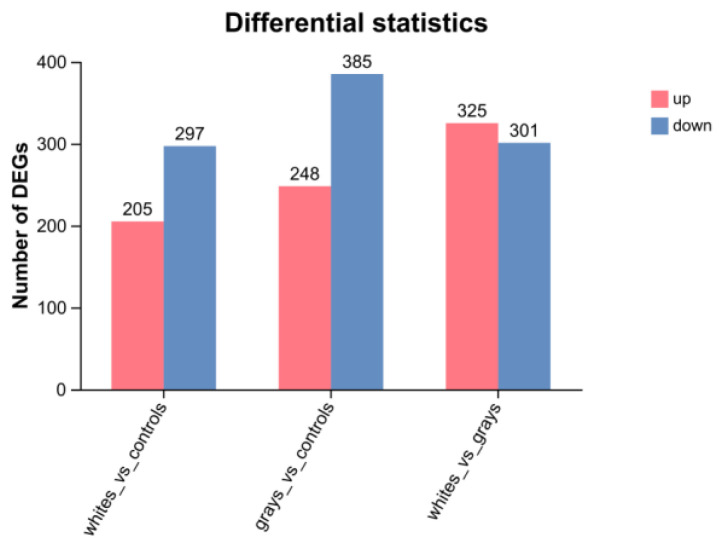
DEGs across color morph comparisons in Yangtze sturgeon. Red and blue indicate upregulated and downregulated transcripts, respectively.

Pairwise comparisons identified extensive transcriptional reprogramming among color morphs. A total of 502 differentially expressed genes (DEGs) between the white and black groups (205 up-, 297 downregulated), 633 DEGs between the gray and black groups (248 up-, 385 downregulated), and 626 DEGs between the white and gray groups (325 up-, 301 downregulated) were identified (Figure 3).

### 2.4. Functional Enrichment Analysis of DEGs

**Figure 4:**
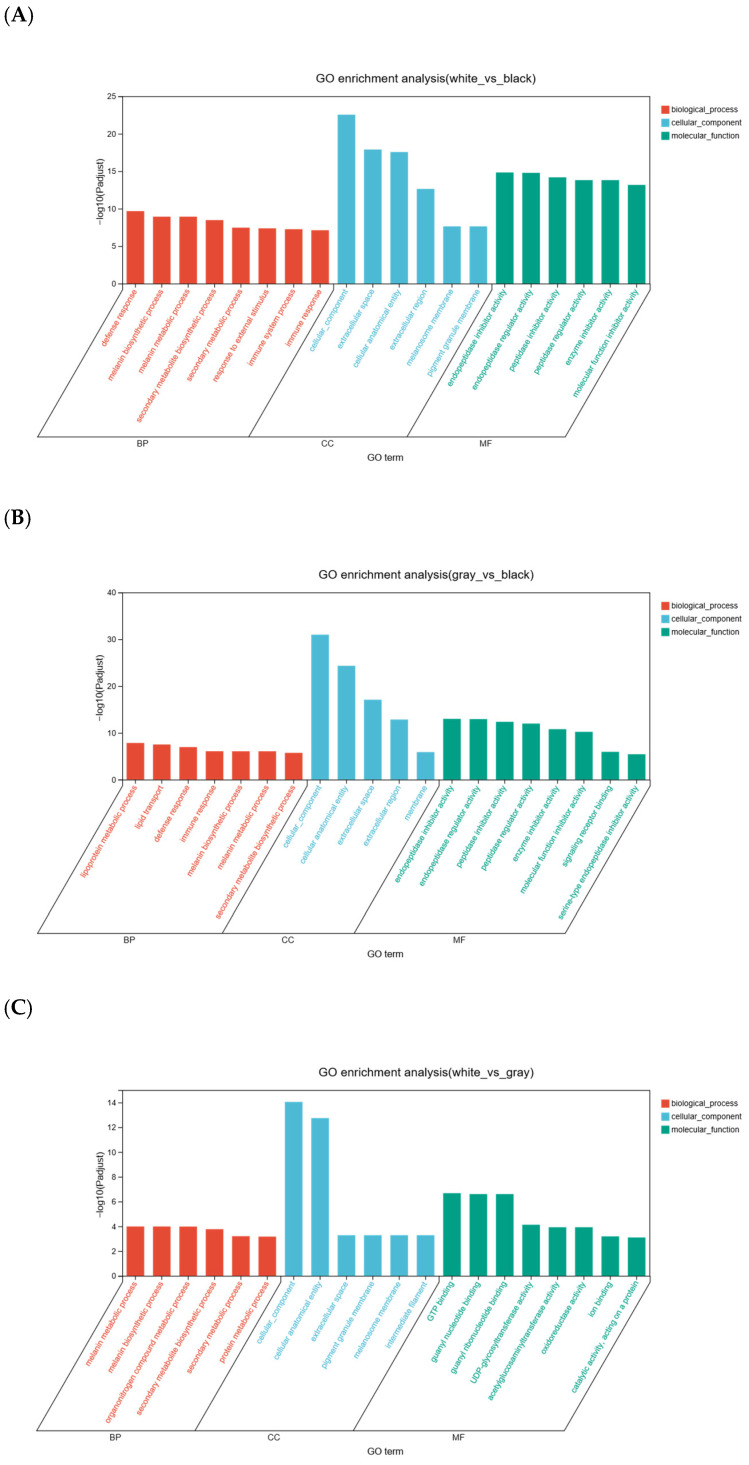
GO enrichment of DEGs among color morph comparisons in Yangtze sturgeon. The top 20 enriched terms are shown for the (**A**) white and black, (**B**) gray and black, and (**C**) white and gray groups.

Gene Ontology (GO) enrichment analysis revealed distinct biological processes associated with color morph differentiation (Figure 4). In the white vs. black comparison, DEGs were significantly enriched in categories linked to defense response, melanin biosynthetic process, immune regulation, and response to external stimuli (Figure 4A). The gray vs. black comparison highlighted enrichment in lipoprotein metabolism, lipid transport, and immune processes (Figure 4B), reflecting metabolic remodeling associated with intermediate pigmentation. The comparisons between the white and gray groups indicated strong enrichment in melanin metabolism, protein processing, and pigment transport pathways (Figure 4C).

### 2.5. KEGG Pathway Enrichment Reveals Convergent Regulation of Pigmentation and Immunity

**Figure 5:**
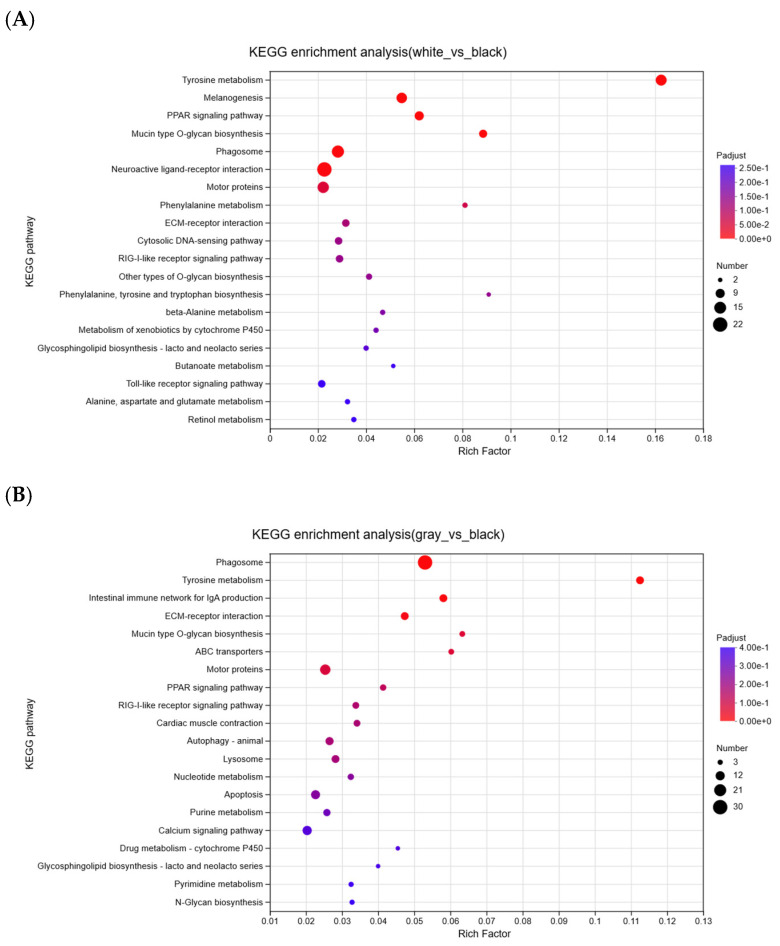

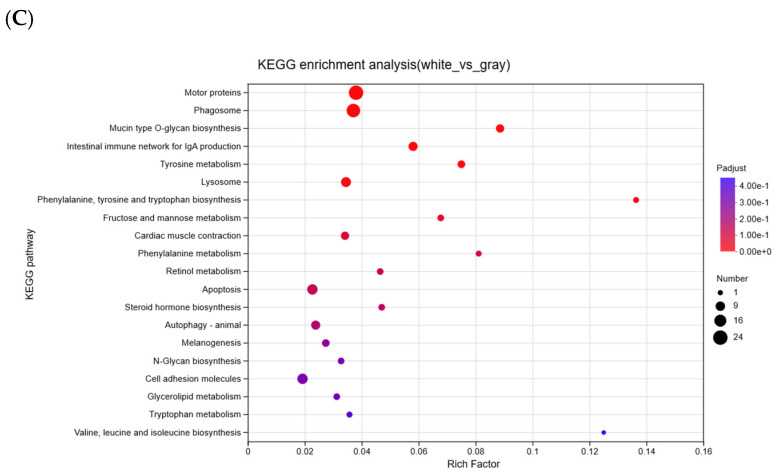
KEGG enrichment of DEGs among color morphs in Yangtze sturgeon. (**A**): KEGG enrichment analysis between the white group and the black group. (**B**): KEGG enrichment analysis between the gray group and the black group. (**C**): KEGG enrichment analysis between the white group and the gray group. The vertical axis represents the pathway name, and the horizontal axis represents the ratio of each factor. The ratio is related to the degree of enrichment, and the size and color of the points represent the number of genes and the *p*-adjust value, respectively.

The Kyoto Encyclopedia of Genes and Genomes (KEGG) analysis revealed that pigmentation loss was associated with strong enrichment of pathways involved in tyrosine metabolism (13), melanogenesis (12), peroxisome proliferator-activated receptor (PPAR) signaling (9), and phagosome activity (7) (Figure 5A). In gray and black comparisons, enriched pathways included phagosome (30), tyrosine metabolism (9), and intestinal immune network for IgA production (9) (Figure 5B). The white and gray comparison similarly revealed enrichment in motor protein activity (24), phagosome formation (21), and tyrosine metabolism (6) (Figure 5C).

### 2.6. Identification of Key Regulatory Genes

From the DEG dataset, 23 pigmentation-related genes were identified (Table 2), including canonical regulators of melanogenesis: *TYR*, *TYRP1*, *DCT*, *MC1R*, *EDNRB*, *ASIP*, *PMEL*, *GPR143*, *MREG*, *MLPH*, *OCA2*, and *SLC45A2*. All exhibited significant downregulation in white sturgeons relative to black individuals, whereas only 11 genes showed parallel patterns in gray vs. black comparisons. This stepwise reduction aligns with the observed phenotypic gradient from full pigmentation (black) to partial (gray) and complete albinism (white). However, four myelin-associated genes (*MBPL*, *MP2PL*, *MPP0*, and *MAG*) were significantly upregulated in white morphs, suggesting compensatory cellular or structural responses within the skin microenvironment (Table 3). Immune-associated transcripts displayed coordinated changes: genes encoding phagosome components (*C3*, *procathepsin L*, *MHC I*, *MHC II*, *TAP2*, and *Ig heavy chain*) were downregulated, while pro-inflammatory mediators (*IL-8L*, *IL-17C*, *CXCL10*, *CCL19*, *CCL20*, *CCR4*, and *AMCF*) were upregulated in white and gray morphs (Table 4). These findings collectively indicate altered expression of immune-related genes in depigmented individuals, characterized by downregulation of phagosome-associated transcripts and upregulation of pro-inflammatory chemokines and cytokines.

**Figure 6:**
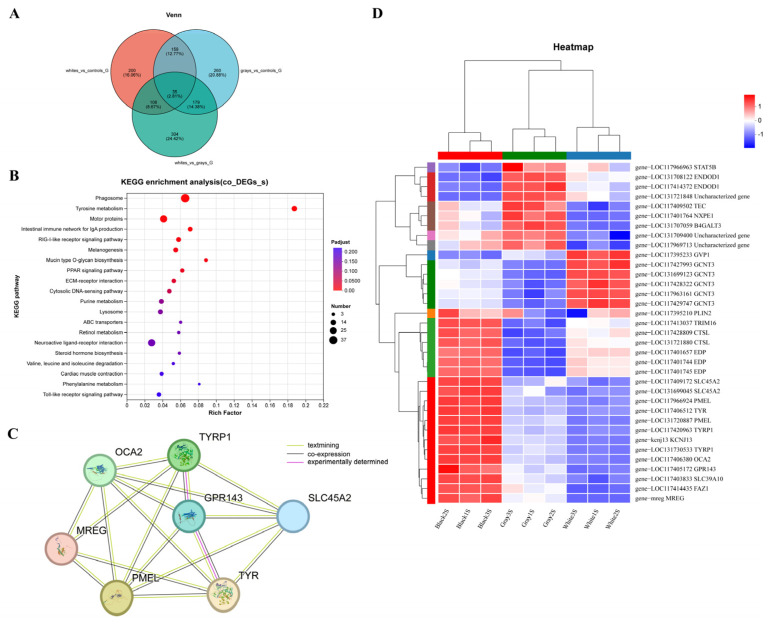
(**A**): Venn analysis of DEGs among different groups in Yangtze sturgeon. Different colored circles represent different gene sets, and the numbers represent the number of common and unique genes between different gene sets. (**B**): Enrichment analysis of common DEGs. The vertical axis represents the pathway name, and the horizontal axis represents the ratio of each factor. The ratio is related to the degree of enrichment, and the size and color of the points represent the number of genes and the *p*-adjust value, respectively. (**C**): PPI network of the DEGs. Nodes represent proteins, and edges indicate predicted or known interactions. (**D**): Clustering analysis of common DEGs. The horizontal axis represents the group, and the vertical axis represents the gene name. Red and blue represent upregulation and downregulation, respectively.

Venn analysis identified 35 common DEGs shared across all three comparisons (Figure 6A). These genes significantly enriched mucin O-glycan biosynthesis, tyrosine metabolism, melanogenesis, and phagosome pathways (Figure 6B). The protein–protein interaction (PPI) network analysis revealed that GPR143 serves as a central hub gene, interacting with multiple other DEGs involved in melanin synthesis (Figure 6C). The hierarchical clustering of these shared DEGs revealed a consistent gradient expression pattern, most notably for *GPR143*, *PMEL*, *TYR*, *TYRP1*, *MREG*, *KCNJ13*, *FAZL*, *SLC45A2*, *SLC39A10*, and *OCA2*, which progressively decreased from black to gray to white morphs (Figure 6C), underscoring their central role in defining pigmentation intensity and associated immune phenotypes.

### 2.7. Validation of DEGs by qRT-PCR

**Figure 7:**
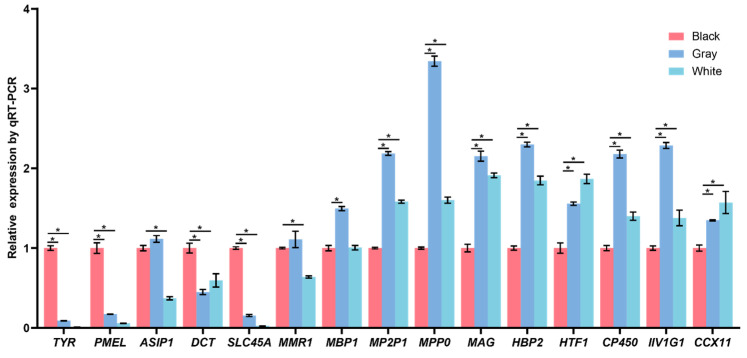
Validation of selected DEGs by qRT-PCR in Yangtze sturgeon. Expression patterns of representative pigmentation- and myelin-associated genes across color morphs are shown. Data are presented as mean ± SEM; statistical significance determined at * *p* < 0.05.

To confirm the RNA-seq results, fifteen representative DEGs were selected for qRT-PCR validation, including melanogenic and myelin-associated genes. The expression profiles of *TYR*, *PMEL*, *DCT*, and *SLC45A2* were significantly downregulated in both white and gray morphs compared with black individuals (Figure 7). However, *MBP1*, *MP2P1*, *MPP0*, and *MAG* were markedly upregulated in white morphs, mirroring transcriptome-derived patterns. The consistency between qRT-PCR and RNA-seq data confirms the robustness and accuracy of the transcriptomic analyses.

### 2.8. Immunofluorescence Staining

**Figure 8:**
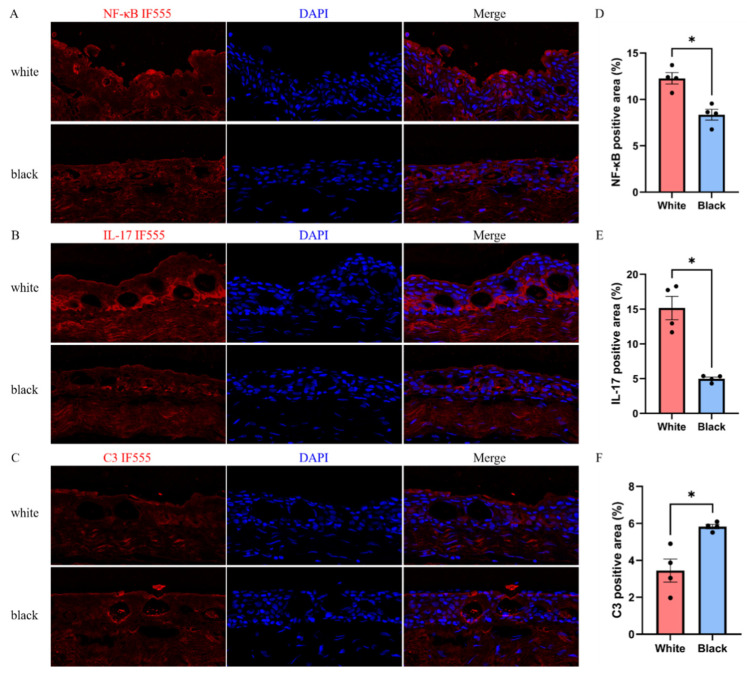
Immunofluorescence staining of NF-κB, IL-17, and C3 in the skin of the white and black groups (*n* = 3 per group). (**A**–**C**) Representative immunofluorescence staining images showing the expression of NF-κB (**A**), IL-17 (**B**), and C3 (**C**) in skin from the white and black groups. Scare bar is 20 μm. Red fluorescence (IF555) represents the target protein, blue fluorescence (DAPI) indicates cell nuclei, and merged images show the combination of both channels. (**D**–**F**) Quantitative analysis of the positive area percentage for NF-κB (**D**), IL-17 (**E**), and C3 (**F**) in each group. Data are expressed as mean ± SEM. * *p* < 0.05 indicates a statistically significant difference between the two groups, which were tested by two-tailed *t*-test.

Due to limited tissue availability from the rare gray morph, immunofluorescence staining was performed only on the white and black groups (not gray). To investigate the expression patterns of inflammatory and complement-related proteins in skin tissues, immunofluorescence staining was performed to visualize NF-κB, IL-17, and C3 in sections from white and black groups (Figure 8), and the results were consistent with the expression levels of the corresponding DEGs identified by the transcriptome. For NF-κB and IL-17, the white group displayed markedly stronger and more extensive positive staining in the epidermis and superficial dermis, whereas the black group exhibited notably diminished signal intensity (Figure 8A,B). Quantitative analysis of positive staining area confirmed these observations; the percentage of NF-κB- and IL-17-positive areas was significantly elevated in the white group relative to the black group (Figure 8D,E). Conversely, complement C3 showed an opposing expression profile. The black group demonstrated stronger and more widespread C3 immunoreactivity, particularly within the epidermal and dermal layers, while the white group displayed comparatively weak C3 staining (Figure 8C). Quantitative analysis further validated this pattern, revealing a significantly higher C3-positive area in the black group compared with the white group (Figure 8F).

**Figure 9:**
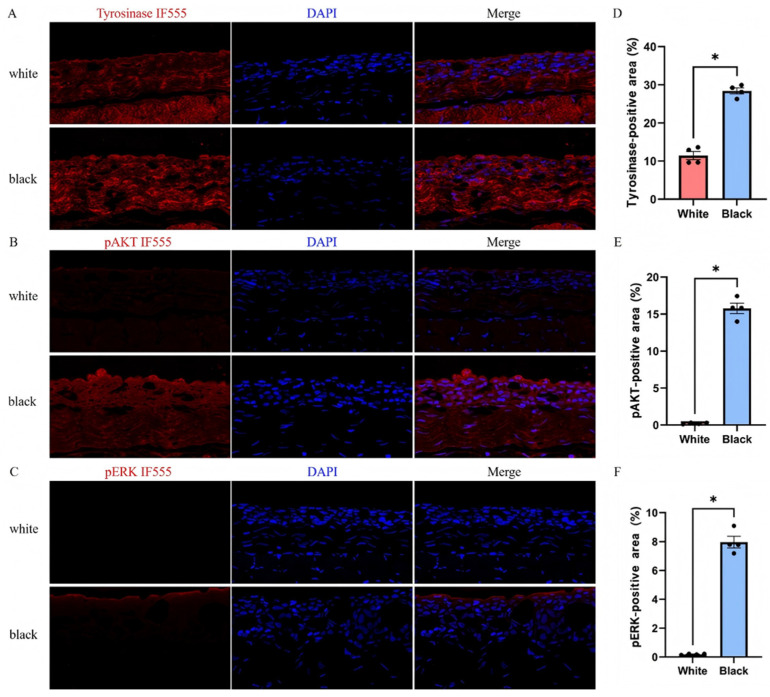
Immunofluorescence staining of TYR, pAKT, and pERK in the skin of the white and black groups. (**A**–**C**) Representative immunofluorescence staining images showing the expression of TYR (**A**), pAKT (**B**), and pERK (**C**) in skin from the white and black groups. Scare bar is 20 μm. Red fluorescence (IF555) represents the target protein, blue fluorescence (DAPI) indicates cell nuclei, and merged images show the overlay of both channels. (**D**–**F**) Quantitative analysis of the positive area percentage for TYR (**D**), p-AKT (**E**), and p-ERK (**F**) in each group. Data are expressed as mean ± SEM. * *p* < 0.05 indicates a statistically significant difference between the two groups, which were tested by two-tailed *t*-test.

To explore the molecular mechanisms underlying skin pigmentation and associated signaling pathway activation, immunofluorescence staining was performed to assess the expression of TYR, pAKT, and pERK in skin from the white and black groups (Figure 9). Consistent with the transcriptome and qRT-PCR, TYR protein expression was markedly elevated in the black group, which exhibited intense and widespread red immunofluorescence throughout the epidermal layers, particularly in the basal and suprabasal regions. In contrast, the white group displayed only faint and sparse positive staining (Figure 9A). Quantitative analysis confirmed that the TYR-positive area was significantly higher in the black group than in the white group (Figure 9D), consistent with enhanced melanin synthesis in pigmented skin.

Similarly, both pAKT and pERK showed substantially elevated immunoreactivity in the black group, with strong signals detected in the epidermis and superficial dermis (Figure 9B,C). In contrast, the white group exhibited negligible p-AKT and p-ERK staining, with only minimal background signal (Figure 9E,F), indicating robust activation of the AKT/ERK signaling cascade in pigmented skin.

## 3. Discussion

**Figure 10:**
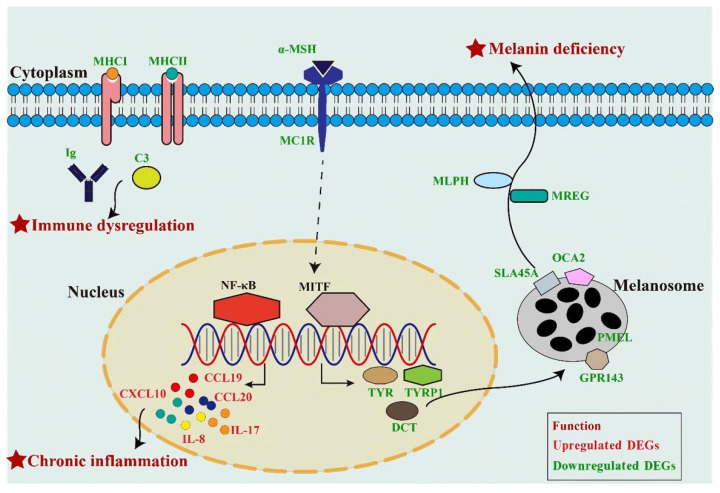
A schematic illustration of skin color formation and immune regulation mechanism of albino Yangtze sturgeon.

Body coloration is a fundamental vertebrate phenotype, integrating pigment synthesis, chromatophore distribution, and complex regulatory networks that jointly shape organismal fitness. Although albinism has been widely reported across mammals, reptiles, and teleost, its genetic and physiological basis remains poorly understood in sturgeons, an ancient lineage that diverged early in ray-finned fish evolution [5,7,8,17]. The Yangtze sturgeon, a “living fossil” at the root of the teleost radiation and now functionally extinct in the wild, represents a pivotal model for understanding both the evolution and the conservation consequences of pigment loss [9]. It is also a first-class national protected animal in China, with its wild population declared extinct by the IUCN [12]. In this study, we combined histological and transcriptomic analyses to uncover the cellular and molecular basis of pigmentation deficiency in white and gray morphs of Yangtze sturgeon. On the basis of this phenotypic evidence, the white morph is referred to as phenotypically albino throughout this study. Genetic confirmation of albinism (e.g., identification of causative mutations in melanogenesis genes) was not performed. Beyond revealing the change in melanin biosynthesis, our findings demonstrate that albinism in this species is intrinsically linked to altered expression of immune-related genes (Figure 10), suggesting that pigmentation loss may have physiological costs directly relevant to the species’ health and reintroduction success.

### 3.1. Molecular Mechanisms Underlying Melanin Deficiency in Albino and Gray Yangtze Sturgeon

The histological examination confirmed the absence of melanin in white skin and a marked reduction in gray skin of the Yangtze sturgeon phenotypes consistent with albinism in other teleosts such as the *Russian sturgeon* (*Acipenser gueldenstaedtii*) [18]. Melanin serves not only as a pigment providing camouflage and photoprotection but also as a multifunctional biopolymer that contributes to detoxification and immune defense [19]. Its biosynthesis is orchestrated by multiple signaling axes, including Wnt, MAPK, AKT, ERK, and the canonical α-MSH-MC1R-MITF cascade, which collectively regulate melanocyte differentiation and the enzymatic activity of tyrosinase family members [20,21,22,23].

In the present study, transcriptomic profiling revealed a pronounced downregulation of the melanogenesis pathway in hypopigmented morphs. Core structural and enzymatic genes, including *PMEL*, *TYR*, *TYRP1*, and *DCT,* as well as regulators of melanosome acidification (*SLC45A2*, *OCA2*) and vesicular trafficking (*MREG*, *MLPH*), were all significantly suppressed. These genes represent conserved modules of vertebrate pigmentation: *PMEL* forms the fibrillar scaffold of melanosomes [24], and its loss in Nile tilapia (*Oreochromis niloticus*) drastically reduces pigmentation [25]; mutations in *TYR* and *TYRP1* abolish melanin formation in zebrafish and yellow catfish [26,27]; and dysfunction of *SLC45A2* or *OCA2* disrupts melanosomal pH homeostasis and maturation [28,29,30]. The consistent downregulation of *MREG* and *MLPH* further implies defects in melanosome transport and deposition, mirroring observations in mlph-deficient zebrafish [31]. Additionally, the low expression of endothelin receptor type B-like (*ERTB*) genes in albino sturgeon aligns with pigment defects observed in piebald mice [32]. In addition, the upregulation of myelin-associated genes (*MBPL*, *MP2PL*, *MPP0*, and *MAG*) in white morphs is intriguing. Melanocytes and Schwann cells share a common neural crest origin [33], and the expression of myelin genes in depigmented skin may reflect a shift in neural crest-derived cell populations or a compensatory glial response. Alternatively, these genes may have uncharacterized functions in skin homeostasis independent of myelination.

Interestingly, unlike findings in many teleosts such as the northern snakehead [15], this study did not observe significant changes in *MITF* gene expression between color morphs, similar to reports in *Russian sturgeon* [18]. This absence of transcriptional change does not preclude the involvement of MITF at post-transcriptional or post-translational levels. Potential explanations include tissue-specific expression dynamics (MITF may be regulated in a spatially restricted manner not captured by whole-skin sampling), modulation via microRNAs targeting *mitf* transcripts, or alterations in protein phosphorylation and stability that affect its transcriptional activity without altering mRNA abundance. This study did not assess the expression of MITF protein; future studies will need to examine MITF at the post-translational level using Western blot. Functional validation through siRNA knockdown or CRISPR/Cas9 approaches will be essential to clarify the precise role of MITF in sturgeon melanogenesis. Importantly, a Venn analysis identified 35 shared differentially expressed genes across comparisons among black, gray, and white morphs. These included key melanogenic regulators (*GPR143*, *PMEL*, *TYR*, *TYRP1*, *MREG*, *SLC45A2*, and *OCA2*), whose expression exhibited a clear gradient from black to gray to white. This gradient strongly supports a model in which pigment intensity reflects progressive failure of a core regulatory ensemble controlling melanosome biogenesis, ion homeostasis, and intracellular transport. This molecular pattern is consistent with findings from other fish species but reveals a broader suppression of melanogenic activity in *A. dabryanus*. In Russian sturgeon, albinism correlates with differential expression of *DCT*, *TYRP1B*, and *SLC45A2* [18], while in turbot (*Scophthalmus maximus*) and red crucian carp (*Carassius auratus* red var.), hyperpigmented skin shows selective upregulation of *TYR*, *TYRP1*, and *OCA2* [6,34]. In contrast, the present dataset reveals a comprehensive downregulation of melanogenesis-related transcripts, suggesting that albinism in the Yangtze sturgeon results from multilayered suppression of pigment synthesis and melanosome maturation rather than single-gene defects. Comparative genomics places sturgeons at the base of the teleost radiation, after two rounds of vertebrate whole-genome duplication (2R-WGD) but before the teleost-specific third round (Ts3R) [9]. Sturgeons and paddlefish share an ancestral whole-genome duplication event followed by independent rediploidization, which may have contributed to the retention of multiple paralogs of pigmentation and immune genes. This integrated repression underscores a potentially unique evolutionary trajectory of pigmentation regulation in sturgeons, one that may reflect basal vertebrate mechanisms distinct from those of derived teleosts. It should be noted that the histological assessments in this study were primarily qualitative in nature, and quantitative morphometric parameters such as melanin granule density, melanocyte number, and mucous cell abundance were not systematically evaluated and thus need to be further determined. It is also worth noting that the number of differentially expressed genes identified between color morphs was relatively modest given the pronounced pigmentation differences. The modest number of DEGs identified may reflect our stringent filtering criteria (|log_2_FC| ≥ 1, FDR < 0.05), which could exclude biologically relevant genes with moderate expression changes, such as *MITF*. This possibility should be considered when interpreting the transcriptional landscape of pigmentation loss. Additionally, causative mutations in pigmentation-related genes (e.g., *TYR*, *OCA2*, and *SLC45A2*) were not screened, leaving the genetic basis of albinism in this species unconfirmed.

### 3.2. Pigmentation Loss Is Accompanied by Broad Immune Dysregulation

The vertebrate skin is not only a physical barrier but also a key immune organ, integrating pigment-producing cells with resident immune networks. From an evolutionary perspective, the tight coupling of pigment and immune pathways in sturgeons may reflect the ancestral neural-crest origin of melanocytes and macrophages, with both cell types utilizing the MITF–CREB–ROS pathway for antimicrobial responses [35,36]. Our study reveals that melanin deficiency in the Yangtze sturgeon coincides with profound substantial changes in the expression of immune-related genes, characterized by lower expression of certain innate defense-related genes and higher expression of certain proinflammatory genes. This dual phenotype mirrors the immunological vulnerability often associated with albinism in other taxa [37]. To directly assess the functional consequences of pigment-associated immune dysregulation, future studies should conduct controlled infection challenges comparing albino, gray, and black morphs. Quantifying survival rates, bacterial loads, and immune gene expression post-infection would provide objective evidence for differential susceptibility and inform conservation strategies.

Genes encoding key elements of pathogen recognition and antigen processing including *complement C3*, *procathepsin L*, *MHC I*, *MHC II*, and *TAP2* were significantly downregulated in albino and gray morphs, indicating a potential inhibitory effect in pathogen recognition, opsonization, and antigen presentation capacity. The observed co-regulation of melanogenic and phagosome-related genes may reflect their shared dependency on lysosome-related organelle biogenesis pathways, as melanosomes and phagosomes utilize overlapping machinery for maturation and intracellular trafficking [38]. In teleosts, these molecules form the backbone of both innate and adaptive immune defense, facilitating phagocytosis, opsonization, and antigen presentation [39]. Their downregulation suggests a transcriptional state that may be associated with reduced immune surveillance capacity, although functional validation is required. Indeed, in the Yangtze sturgeon and Chinese sturgeon, *MHC I* and *MHC II* expression is normally upregulated during bacterial challenge [40,41], underscoring that the hypoexpression observed here reflects functional compromise rather than baseline variability. This profile parallels the lysosomal and immune defects seen in mammalian Chediak–Higashi syndrome, where melanosome-related abnormalities are mechanistically linked to impaired phagolysosomal activity [42].

The complement system, a key axis of innate immunity in bony fishes, was similarly attenuated. Downregulation of C3 and related components likely reduces phagocytic efficiency and delays immune activation, as evidenced by studies showing that recombinant C3 enhances macrophage function and pathogen resistance in tilapia and flounder [43,44]. Such suppression in albino sturgeons may therefore increase susceptibility to microbial infection, a critical liability for individuals released into wild environments.

Paradoxically, this immunosuppression coexists with chronic inflammatory activation. Hypopigmented morphs exhibited significant upregulation of inflammatory cytokines and chemokines, including IL-8, IL-17, CXCL10, CCL19, and CCL20. Under physiological conditions, interleukins and chemokines mobilize immune cells within skin tissue to counteract pathogens in a transient manner, resolving to a steady state after pathogen clearance, a process exemplifying the dynamic nature of inflammation [45]. However, in hypopigmented Yangtze sturgeons, this response appears excessive and persistent, escalating into a chronic state known to impede tissue repair [46]. This chronic inflammatory state finds parallels in mammalian models; for instance, albino mice exhibit more pronounced inflammatory cell infiltration, scab formation, and elevated myeloperoxidase activity after UV exposure compared to pigmented counterparts [2]. The persistent elevation of IL-17 is particularly notable, given its dual role in driving keratinocyte activation and oxidative stress–mediated melanocyte damage [47]. This cytokine milieu likely establishes a self-sustaining cycle in which inflammatory stress exacerbates melanocyte dysfunction, while pigment loss amplifies vulnerability to UV-induced damage and oxidative injury [48]. Indeed, the loss of MC1R-mediated signaling, which normally restrains inflammation through NF-κB suppression, could further contribute to the chronic inflammatory phenotype observed in albino individuals [46,49]. However, immunofluorescence validation for the gray morph was not possible due to sample scarcity, meaning this needs to be further explored. Structurally, the looser dermis in hypopigmented morphs could hypothetically facilitate antigen exposure, driving the inflammatory response [50], whereas the compact dermis of black fish may limit pathogen entry, permitting a more regulated immune state. Such persistent inflammation, coupled with weakened phagocytic and complement responses, defines a transcriptional state that may reflect altered immune readiness.

The convergence of pigmentation loss and immune alteration in *A. dabryanus* suggests potential considerations for conservation management that warrant further investigation. In aquatic ecosystems, melanin contributes not only to visual camouflage but also to UV protection, antioxidant defense, and pathogen resistance. It is plausible that the loss of melanin-associated functions could reduce their ecological fitness, but this hypothesis requires direct testing through survival and pathogen resistance studies. If pigmentation is considered as a potential management criterion in the future, reintroduction programs may need to evaluate whether pigmentation morphs differ in survival, pathogen resistance, and environmental tolerance. At present, such an application remains a hypothesis requiring direct testing. If future studies confirm that pigment deficiency is associated with measurable physiological costs, targeted interventions such as supplementation with immunostimulants (e.g., β-glucans, vitamins C and E) or provision of enhanced UV protection could be explored as potential management measures for albino and gray individuals. Furthermore, the emergence of albino and gray morphs within captive populations raises potential concerns regarding genetic drift and inbreeding, both of which can erode adaptive potential in small, isolated populations. From a mechanistic standpoint, our integrated histological and transcriptomic analyses delineate how pigmentation loss in the Yangtze sturgeon reflects a coordinated alteration of melanin biosynthesis and a correlated shift in immune-related gene expression, rather than a demonstrated collapse of immune function. The concurrent downregulation of melanogenic and immune-related transcripts suggests a potential physiological interconnection between pigmentation and immune competence at the molecular level, an axis of vertebrate biology that remains underexplored in basal fish lineages. These findings thus provide not only a molecular basis for albinism in *A. dabryanus* but also a conceptual framework for understanding how pigmentation, immunity, and fitness intersect during evolution and conservation. Importantly, the current study identifies correlations rather than causal relationships. Functional experiments (e.g., gene knockdown or pathogen challenge) are required to determine whether melanin deficiency directly drives immune-related molecular changes or whether these phenotypes arise from shared genetic or developmental pathways. 

## 4. Materials and Methods

### 4.1. Experimental Animals and Ethical Approval

The white, gray, and black color morphs of Yangtze sturgeon juveniles were obtained as the second generation offspring from Fisheries Institute, Sichuan Academy of Agricultural Sciences (SAAS, Chengdu, China) to ensure a uniform genetic background. Induced breeding of adult male and female with normal black pigmentation was performed on 27 March 2025, followed by artificial insemination. All individuals used in this study originated from the same artificial spawning batch to minimize genetic background variation. Fertilized eggs were cultured under controlled hatchery conditions, and larvae were reared to the juvenile stage after hatching on 1 April 2025. The juvenile Yangtze sturgeon was reared in the same tank system in flow-through filtered river water (19.5 ± 0.5 °C) with dissolved oxygen (DO) level > 6 mg/L under natural light. At the juvenile stage, fish were fed with artificial feed (Shengsuo, Yantai, China) about four times a day at 3:00, 9:00, 15:00, and 21:00 until a little residual bait remained. 

All experimental protocols were reviewed and approved by the Institutional Animal Care and Use Committee (IACUC) of the Sichuan Academy of Agricultural Sciences (Approval No. 20250614001A) and adhered to institutional and national guidelines for animal welfare. Prior to sampling, fish were euthanized humanely with an overdose of buffered MS-222 (80 mg/L) to minimize distress.

### 4.2. Samples Collection

The 27 fish representing three color morphs (*n* = 9 per color) were anesthetized with MS-222, and biometric parameters (body weight and total length) were recorded. Each individual provided one skin sample. These 9 samples of each color were used for histology and gene expression analysis. For transcriptome analysis, three pools per color were used, each pool comprising skin from three individuals. For immunofluorescence (IF) staining, due to tissue availability, we used 3 individuals per group (white and black only). Skin samples were collected from a standardized location along the lateral line using sterile surgical scalpels. The excised tissues were immediately flash-frozen in liquid nitrogen and stored at −80 °C for molecular analyses. Parallel samples containing skin and underlying muscle were fixed in 4% paraformaldehyde in PBS (pH 7.4) (Servicebio, Wuhan, China) for histological and melanin-specific staining.

### 4.3. Histological and Morphological Analyses

Skin tissue samples were fixed in 4% paraformaldehyde (Servicebio, China) for 24 h at 4 °C, dehydrated using graded ethanol, cleared in xylene, and embedded in paraffin. Serial 5 µm sections were prepared using a rotary microtome. For general histological assessment, sections were deparaffinized, rehydrated, and stained with hematoxylin and eosin (H&E) following standard protocols. Briefly, slides were differentiated in acid ethanol, blued in ammonia water, counterstained with eosin, and mounted in neutral balsam. For melanin visualization, adjacent serial sections were processed using the Fontana–Masson staining method (Solarbio, China). After deparaffinization and rehydration, sections were incubated in ammoniacal silver solution at 60 °C in the dark until satisfactory chromogenic intensity was reached. The reaction was stopped with hot distilled water, followed by nuclear counterstaining with nuclear fast red. Stained slides were examined and digitally imaged using a Leica stereomicroscope to document tissue morphology and pigment distribution.

### 4.4. RNA Extraction, Library Construction, and Transcriptome Assembly

Total RNA was extracted from tissue samples using TRIzol® reagent (Invitrogen, Carlsbad, CA, USA) according to the manufacturer’s protocol. RNA quality was verified using Agilent 5300 Bioanalyzer (Agilent Technologies, Santa Clara, CA, USA), and concentrations were determined with a NanoDrop 2000 spectrophotometer (Thermo, Wilmington, NC, USA). Only samples meeting the following quality criteria were used for sequencing: total RNA ≥ 2.0 µg, RNA Quality Number (RQN) ≥ 4.5. RNA purification, reverse transcription, library preparation, and sequencing were conducted by Shanghai Meiji Biotechnology Co., Ltd. (Shanghai, China). mRNA was isolated from total RNA using oligo(dT) magnetic beads and fragmented for cDNA synthesis. First-strand and second-strand cDNA were synthesized using random hexamer primers. The resulting cDNA was subjected to end repair, A-tailing, and adapter ligation. Libraries (300–400 bp) were size-selected using magnetic beads and PCR-amplified (10–15 cycles). The raw paired-end reads were processed using fastp to remove adaptors and low-quality sequences. Then, the high-quality reads were aligned to the reference genome using HISAT2, and transcript assembly was performed with StringTie to generate sample-specific transcriptomes.

### 4.5. Differential Expression Analysis and Functional Enrichment Analysis

The gene expression levels were quantified using RSEM version 1.3.3. Differential expression analysis was performed using DESeq2 (version 1.30.0), with raw read counts as input. Regarding transcript redundancy, we used StringTie version 2.1.1 with default parameters, and multiple testing correction was performed using the Benjamini–Hochberg (BH) false discovery rate (FDR) method with a threshold of *p*-adjust < 0.05. Differentially expressed genes (DEGs) were defined by |log_2_(fold change)| ≥ 1 and *p*-adjust < 0.05. For visualization purposes only (PCA plots and heatmaps), expression values were normalized to Transcripts Per Million (TPM). Functional enrichment analyses were conducted to identify Gene Ontology (GO) terms and KEGG pathways significantly associated with DEGs. GO enrichment was performed using Goatools (https://github.com/tanghaibao/Goatools (accessed on 23 August 2025)), with false discovery rate (FDR) correction at *p* < 0.05. KEGG pathway enrichment was analyzed using the scipy statistical package in Python (version 3.14), applying Bonferroni correction (*p* < 0.05). The top 20 significantly enriched pathways were visualized as bubble plots using the dotplot function.

### 4.6. qRT-PCR Analysis

To validate the transcriptome sequencing data, we selected the DEGs related to pigmentation and immune regulation and quantified them by quantitative real-time PCR (qRT-PCR). Total RNA extraction and quality assessment were performed as described above. Complementary DNA (cDNA) was synthesized from the high-quality RNA using the PrimerScriptTM RT Reagent Kit (Takara, Dalian, China). Target genes were amplified using gene-specific primers designed from transcriptome data. *β-actin*, previously validated as a stable reference gene in *A. dabryanus* [51], was used for normalization. There was no significant difference in the expression level of the *β-actin* gene among different colored skin. All reactions were performed in triplicate according to established laboratory protocols [52]. All primer amplification efficiencies ranged from 96.6% to 100.1%, with correlation coefficients (R^2^) between 0.961 and 0.998, confirming quantitative reliability. Relative gene expression levels were calculated using the 2^−ΔΔCt^ method [53]. The primer sequences are listed in Table 5.

### 4.7. Immunofluorescence Staining and Quantitative Analysis

Formalin-fixed (4% paraformaldehyde in PBS, pH 7.4), paraffin-embedded skin sections were deparaffinized, rehydrated, and subjected to antigen retrieval by heating in citrate buffer (10 mM, pH 6.0) at 95 °C for 20 min. After cooling to room temperature, sections were blocked with 5% normal goat serum in PBS containing 0.3% Triton X-100 for 1 h at room temperature. The sections were then incubated overnight at 4 °C with primary antibodies against NF-κB (Bioss, Bs-23303R, 1:200), IL-17 (BUABIO, ER1706-91, 1:200), C3 (ZENBIO, R23673, 1:200), TYR (ZENBIO, R381782, 1:100), pAKT (CST, 9018S, 1:100), and pERK (Servicebio, GB1104, 1:100), followed by incubation with HRP-labeled goat anti-rabbit IgG secondary antibodies (Servicebio, GB23303, 1:500). Cell nuclei were counterstained with DAPI (BEYOTIME, C1002). Fluorescence images were captured using a fluorescence microscope (NIKON ECLIPSE TI-SR (NIKON, Tokyo, Japan)), and the percentage of the positive area for each target protein was quantified using ImageJ software version 1.8.0 [54]. Image acquisition and quantification were performed blinded to the group allocation. Thresholds for positive area quantification were set automatically using ImageJ’s default algorithm and then applied uniformly across all images from the same staining run. Negative controls were performed by omitting the primary antibody on adjacent sections; no specific fluorescence signal was detected under these conditions. HRP-labeled secondary antibodies were detected using tyramide signal amplification with a fluorescent tyramide substrate (IF555), which converts HRP activity into a fluorescent signal.

### 4.8. Statistical Analysis

Data are expressed as mean ± standard error of the mean (SEM). Prior to parametric testing, normality was assessed using the Shapiro–Wilk test, and the homogeneity of variances was assessed using Levene’s test. For histological and qRT-PCR comparisons, one-way ANOVA followed by Tukey’s HSD post hoc test was performed using SPSS 26.0. For two-group comparisons (IF), independent two-tailed Student’s *t*-test was used. *p* < 0.05 indicates a significant difference.

## 5. Conclusions

This study provides the first comprehensive molecular characterization of pigmentation loss in the Yangtze sturgeon, integrating histological evidence with transcriptomic profiling across black, gray, and albino morphs. We show that melanin deficiency arises from widespread suppression of melanogenesis-related genes controlling melanosome formation, pigment synthesis, and intracellular transport. Concurrently, pigment loss is associated with impaired immune pathways and chronic inflammatory activation, reflecting physiological alteration in the skin. These results reveal a correlative coupling between pigmentation and immune regulation in a basal vertebrate lineage and highlight the potential vulnerabilities of albino individuals in conservation programs. Taken together, they underscore the need to incorporate molecular phenotyping into breeding and reintroduction strategies to safeguard the adaptive resilience of this critically endangered species.

## Figures and Tables

**Table 1:** Quality control of transcriptome sequencing of Yangtze sturgeon skin.

Sample	Clean Reads	Clean Bases	Error Rate (%)	Q20 (%)	Q30 (%)	GC Content (%)	RQN
White1S	42,570,316	6,390,990,887	0.0126	98.85	94.86	47.82	9.1
White2S	42,810,164	6,428,646,641	0.0127	98.82	94.65	47.84	9.4
White3S	38,314,446	5,753,084,785	0.0127	98.81	94.66	47.69	9.2
Gray1S	43,034,504	6,463,788,652	0.0126	98.87	94.86	47.66	8.8
Gray2S	40,573,754	6,094,222,415	0.0127	98.82	94.68	47.32	8.3
Gray3S	36,336,158	5,458,884,115	0.0128	98.78	94.41	48.06	9.7
Black1S	39,378,076	5,910,956,122	0.0128	98.79	94.5	47.43	9.1
Black2S	38,733,598	5,810,931,430	0.0127	98.82	94.69	47.71	9.3
Black3S	38,803,880	5,825,196,430	0.0127	98.81	94.65	48.02	9.3

**Table 2:** DEG mining related to body color regulation in Yangtze sturgeon.

	Gene ID	Gene Name	White_vs._Black log2FC	Sig	Gray_vs._Black log2FC	Sig
Melanin synthesis	LOC117405438	*tyrosinase*	−7.01	yes	−5.33	yes
	LOC117406512	*tyrosinase-like*	−7.16	yes	−4.42	yes
	LOC117962704	*tyrosinase-like*	−5.84	yes	−3.79	no
	LOC117965811	*tyrosinase-like*	−4.88	yes	−3.74	no
	LOC117414288	*tyrosinase-like*	−6.22	yes	−2.66	no
	LOC117420963	*5,6-dihydroxyindole-2-carboxylic acid oxidase-like*	−6.26	yes	−3.33	yes
	LOC131730533	*5,6-dihydroxyindole-2-carboxylic acid oxidase-like*	−5.87	yes	−2.90	yes
	LOC117408362	*5,6-dihydroxyindole-2-carboxylic acid oxidase-like*	−1.13	yes	−0.14	no
	*DCT*	*dopachrome tautomerase*	−2.18	yes	−1.88	yes
	LOC117406380	*P protein*	−6.54	yes	−3.14	yes
	LOC117972966	*P protein-like*	−5.93	yes	−4.79	no
Development and differentiation of pigment cells	LOC117425852	*melanocyte-stimulating hormone receptor-like*	−2.35	yes	−0.53	no
	LOC117430294	*endothelin receptor type B-like*	−1.18	yes	−0.58	no
	*ASIP1*	*agouti signaling protein 1*	−1.06	yes	0.26	no
melanosome transport and localization	LOC117426190	*melanophilin-like*	−1.43	yes	−1.25	yes
	LOC117405668	*melanophilin-like*	−0.99	no	−1.36	yes
	LOC117409172	*membrane-associated transporter protein*	−4.62	yes	−2.76	yes
	*MREG*	*melanoregulin*	−9.63	yes	−1.83	yes
Genes related to signal transduction and regulation	LOC117966924	*melanocyte protein PMEL-like*	−5.72	yes	−3.94	yes
	LOC131720887	*melanocyte protein PMEL-like*	−6.32	yes	−3.73	yes
	LOC117406619	*G-protein coupled receptor 143-like*	−2.33	yes	−1.28	yes
	LOC117405172	*G-protein coupled receptor 143-like*	−2.71	yes	−1.19	yes
	*SLC24A5*	*solute carrier family 24 member 5*	−3.97	yes	−0.78	no

**Table 3:** DEG mining related to cellular structural maintenance in Yangtze sturgeon.

Gene ID	Gene Name	White_vs._Black log2FC	Sig	Gray_vs._Black log2FC	Sig
LOC117400640	*myelin basic protein*	1.04	yes	0.83	no
LOC117394398	*myelin P2 protein*	1.10	yes	0.63	no
LOC117410179	*myelin protein P0*	1.24	yes	0.88	no
LOC117966809	*myelin protein P0*	1.03	yes	0.58	no
LOC117433593	*myelin-associated glycoprotein*	1.02	yes	0.17	no

**Table 4:** DEG mining related to immune defense.

Function	Gene ID	Gene Name	White_vs._Black log2FC	Sig	Gray_vs._Black log2FC	Sig
phagosome signaling pathway	LOC117409788	*complement C3*	−2.37	yes	−1.85	yes
	LOC131706606	*complement C3-like*	−2.54	yes	−1.87	yes
	LOC117398833	*complement C3-like*	−4.49	yes	−4.31	yes
	LOC131740028	*procathepsin L-like*	−0.97	no	−2.16	yes
	LOC117401272	*procathepsin L-like*	−0.18	no	−2.31	yes
	LOC117401377	*procathepsin L-like*	−0.14	no	−1.73	yes
	LOC117428809	*procathepsin L-like*	−1.00	yes	−2.35	yes
	LOC117428941	*procathepsin L-like*	−0.40	no	−1.52	yes
	LOC117966330	*procathepsin L-like*	−0.31	no	−1.75	yes
	LOC131721880	*procathepsin L-like*	−1.10	yes	−2.40	yes
	LOC117964886	*H-2 class I histocompatibility antigen, Q9 alpha chain-like*	−0.47	no	−1.11	yes
	LOC117968747	*H-2 class II histocompatibility antigen, A-Q alpha chain-like*	−1.11	yes	−0.89	no
	LOC131709127	*H-2 class I histocompatibility antigen, Q9 alpha chain-like*	−1.01	yes	0.09	no
	LOC131740170	*major histocompatibility complex class I-related gene protein-like*	−0.94	no	−1.93	yes
	LOC131709086	*BOLA class I histocompatibility antigen, alpha chain BL3-7-like*	−1.25	yes	−1.67	yes
	LOC131706958	*antigen peptide transporter 2-like*	−0.30	no	−2.65	yes
	LOC117433950	*antigen peptide transporter 2*	−0.18	no	−1.25	yes
	LOC117398064	*Ig mu chain C region-like*	−0.48	no	−1.92	yes
	LOC131723275	*Ig heavy chain V region 914-like*	−0.49	no	−2.13	yes
	LOC131723276	*Ig heavy chain V region 914-like*	−0.02	no	−1.23	yes
	LOC131723277	*Ig heavy chain V region 6.96-like*	−0.74	no	−2.51	yes
immune response	LOC117394321	*C-C chemokine receptor type 4*	1.17	no	1.51	yes
	*CXCR4A*	*chemokine (C-X-C motif) receptor 4a*	−0.08	no	−1.05	yes
	LOC117403926	*C-X-C motif chemokine 10-like*	2.52	yes	2.60	yes
	LOC117403596	*C-X-C motif chemokine 10-like*	−5.50	yes	0.20	no
	LOC117403923	*C-X-C motif chemokine 11-1-like*	0.77	no	1.26	yes
	LOC117403941	*C-X-C motif chemokine 11-1-like*	1.61	yes	0.88	no
	LOC131697915	*C-X-C motif chemokine 11-1-like*	0.63	no	1.36	yes
	LOC131702573	*C-X-C motif chemokine 11-1-like*	1.51	yes	1.16	no
	LOC117403594	*C-X-C motif chemokine 11-1-like*	1.44	yes	0.73	no
	LOC117969846	*C-C motif chemokine 19-like*	1.01	yes	0.78	no
	LOC117416555	*C-C motif chemokine 20-like*	0.89	no	1.16	yes
	LOC117432902	*interleukin-2 receptor subunit beta-like*	0.05	no	−1.06	yes
	LOC117420409	*interleukin-8-like*	1.68	yes	1.12	no
	LOC117421523	*interleukin-8-like*	−0.69	no	−2.14	yes
	*IL-17C*	*interleukin-17c*	0.42	no	1.96	yes
	*CLCF1*	*cardiotrophin-like cytokine factor 1*	−1.06	yes	−1.02	yes
	LOC117403838	*alveolar macrophage chemotactic factor-like*	1.91	no	3.44	yes

**Table 5:** Quality control of transcriptome sequencing.

Primer Name	Sequence (5′-3′)	Tm (℃)	Amplification Efficiencies (%)	Product Size (bp)
*TYR*-F	AGCACGGAGCACCTTACACAAC	60	99.5	24
*TYR*-R	AGCACGACTGACAAGACCAAGATG			
*PMEL*-F	CATGGAGGTGGTGGTGTATCA	61.5	99.2	245
*PMEL*-R	TGCTTGGTTCAGGTCGTTCA			
*ASIP1*-F	AACGAGAAGAGAAGCAGTCAGAGAC	59	97.5	222
*ASIP1*-R	AGGCACAGTGTTCACAGCAGAC			
*DCT*-F	GCCTTCATTACCTGGCACAGATACC	60	99.9	286
*DCT*-R	TTCTCCTCAGCACACCTTCATTGG			
*SLC45A2*-F	ACTGAGCGAAGCCAATTCCATTACA	59	96.6	136
*SLC45A2*-R	GTGACTGACACAGAGACAGCGATAG			
*MMR1*-F	GAACACCACACCAACCTCATCAGT	59.5	100.1	231
*MMR1*-R	GCCATAGTCATTCCATCCACCAGTC			
*MBPL*-F	GGACCAAGCCAAACATCTGAACCA	60.6	99.8	165
*MBPL*-R	GGAGTCTGTCACCGCTGTCTTCT			
*MP2PL*-F	TCAATCGTGGGATGGCAAACAGA	58.6	99.2	108
*MP2PL*-R	TTCTCATAGGTCCTTGTGGCAACA			
*MPP0*-F	GGACCATTCAGAGACCGCTTGG	60.9	97.6	106
*MPP0*-R	CGCAGGTGAAGGTTCCGTTGT			
*MAG*-F	CATCAGCAACATAGTGGCGTCAGA	60	96.8	281
*MAG*-R	GGCTCTCCGTCTCGTTGATTGTG			
*HBP2*-F	GGTGCTGCTGGCTTCTGAACAA	61	98	298
*HBP2*-R	TGTGCTGTCGGCTGTGGGATAT			
*HTFL*-F	AAAGCCTCCACTCCCGCCAA	61.7	98	288
*HTFL*-R	AGATCCAGCCAGAAGCCACAGA			
*CP450*-F	CAGTCCACAGTCAGAGCGTTCTC	60	99.4	242
*CP450*-R	TTGTTCCTGCCAACCACCGATT			
*LIVLG1*-F	AATCCACCAATGGCACCTCCTAATC	59.5	98.2	81
*LIVLG1*-R	TGTCACAGAACAGAGCAGCATCC			
*CXC11*-F	GCAACGCTTGTCAACGGCAT	59.6	99.5	121
*CXC11*-R	CACACTTCGGACTCATAGGAAACAC			
*β-actin*-F	GCCCCACCTGAGCGTAAAT	60	98.2	92
*β-actin*-R	TCCTGCTTGCTGATCCACAT			

## Data Availability

The raw data supporting the conclusions of this article will be made available by the authors on request. The raw sequencing data have been deposited in the NCBI Sequence Read Archive (SRA) under BioProject accession number PRJNA1389687 and are publicly available at https://www.ncbi.nlm.nih.gov/bioproject/PRJNA1389687 (accessed on 16 December 2025).
